# Enzyme-Free Detection of Mutations in Cancer DNA Using Synthetic Oligonucleotide Probes and Fluorescence Microscopy

**DOI:** 10.1371/journal.pone.0136720

**Published:** 2015-08-27

**Authors:** Laura Miotke, Arindam Maity, Hanlee Ji, Jonathan Brewer, Kira Astakhova

**Affiliations:** 1 The Division of Oncology, Stanford School of Medicine, Stanford University, Stanford, California, United States of America; 2 Nucleic Acid Center, Department of Physics, Chemistry and Pharmacy, University of Southern Denmark, Odense, Denmark; 3 Memphys Center for Biomembrane Physics, Department of Biochemistry and Molecular Biology, University of Southern Denmark, Odense, Denmark; 4 Dr B C Roy College of Pharmacy and AHS, Durgapur, West Bengal, India; Pennsylvania State Hershey College of Medicine, UNITED STATES

## Abstract

**Background:**

Rapid reliable diagnostics of DNA mutations are highly desirable in research and clinical assays. Current development in this field goes simultaneously in two directions: 1) high-throughput methods, and 2) portable assays. Non-enzymatic approaches are attractive for both types of methods since they would allow rapid and relatively inexpensive detection of nucleic acids. Modern fluorescence microscopy is having a huge impact on detection of biomolecules at previously unachievable resolution. However, no straightforward methods to detect DNA in a non-enzymatic way using fluorescence microscopy and nucleic acid analogues have been proposed so far.

**Methods and Results:**

Here we report a novel enzyme-free approach to efficiently detect cancer mutations. This assay includes gene-specific target enrichment followed by annealing to oligonucleotides containing locked nucleic acids (LNAs) and finally, detection by fluorescence microscopy. The LNA containing probes display high binding affinity and specificity to DNA containing mutations, which allows for the detection of mutation abundance with an intercalating EvaGreen dye. We used a second probe, which increases the overall number of base pairs in order to produce a higher fluorescence signal by incorporating more dye molecules. Indeed we show here that using EvaGreen dye and LNA probes, genomic DNA containing *BRAF* V600E mutation could be detected by fluorescence microscopy at low femtomolar concentrations. Notably, this was at least 1000-fold above the potential detection limit.

**Conclusion:**

Overall, the novel assay we describe could become a new approach to rapid, reliable and enzyme-free diagnostics of cancer or other associated DNA targets. Importantly, stoichiometry of wild type and mutant targets is conserved in our assay, which allows for an accurate estimation of mutant abundance when the detection limit requirement is met. Using fluorescence microscopy, this approach presents the opportunity to detect DNA at single-molecule resolution and directly in the biological sample of choice.

## Introduction

Single-nucleotide polymorphisms (SNPs) and variants (SNVs) are the main source of genetic variation in human genome and other species [1]. Therefore, reliable detection of SNPs and SNVs is an important clinical and translational research tool. Some of the many applications include drug-resistance analysis in viral genomes and cancer associated somatic DNA alterations [2]. One human cancer gene in particular, *BRAF* or v-Raf murine sarcoma viral oncogene homolog B, encodes the protein B-Raf [3]. More formally known as serine/threonine-protein kinase B-Raf, this protein affects intracellular signalling and is involved in directing cell growth [3]. Mutations in *BRAF* have been implicated in some human cancers, particularly melanoma [4]. Incidentally, several drugs have been developed that treat cancers driven by *BRAF*, two of which, vemurafenib and dabrafenib, are now FDA approved for treatment of late-stage melanoma [5,6]. Current detection methods of mutations in *BRAF* subjects biopsy samples to PCR and/or sequencing. A less invasive approach detects *BRAF* mutations directly in circulating tumor DNA (ctDNA) obtained from patient’s plasma [7]. However, since ctDNA are present at very low concentrations (100–300 molecules per 100 μL analyte), highly sensitive and specific detection techniques have to be applied [8].

Generally, development in the field of SNP/SNV diagnostics goes simultaneously in two directions: 1) high-throughput methods, and 2) portable, easy-to-handle assays [8,9]. High-throughput sequencing and polymerase-chain reaction (PCR) are current methods of choice for research on cancer and infectious diseases in developed countries [9]. More available techniques for rapid point-of-care diagnostics in the absence of sequencing and PCR are appealing. Moreover, all high-throughput techniques developed to date apply enzymes in order to achieve the required sensitivity and specificity of detection [9]. Enzymes increase the risk of errors during the analysis and affects stoichiometry of the targeted mutation with respect to wild-type analogue [10]. Thus, all methods of nucleic acids detection and quantification would benefit from alternative enzyme-free strategies [9,10].

An ideal, easy-to-handle diagnostic of SNP/SNV is a simple robust assay with minimal steps, high sensitivity and repeatability at low cost [9]. With these objectives in mind, a solid support or in solution system using optical and electrochemical methods is ideal [11]. Fluorescence is a convenient optical detection method which is broadly applied in both modern state-of-the-art and portable diagnostic assays [9]. In particular, recent developments in fluorescent microscopy is having a huge impact on detection of biomolecules *in vitro* and *in vivo* [12,13]. Microscope techniques have made previously unachievable single-molecule detection available. This provides an opportunity to avoid enzymes when detecting biomolecules *in vitro* and a chance to monitor targets *in vivo* [9–13].

To detect SNP/SNVs at very low concentrations, highly specific and sensitive probes have to be applied. One approach is to place locked nucleic acids (LNAs) with directly attached fluorophores into short oligonucleotides [14]. These types of LNA/DNA capture probes are currently applied in enzymatic genotyping of SNPs in a microarray format and PCR-based techniques [15]. Recently we introduced fluorescently labelled LNA derivatives as a promising tool in advanced genotyping, for example drug-resistance testing of highly polymorphic HIV-1 protease [16]. However, synthesis of fluorescently labelled probes is more expensive and labor-intensive than is necessary for point-of-care applications. A more straightforward probe design could apply the highly efficient hybridization properties of LNA/DNA oligonucleotides in conjunction with a robust, fluorescent intercalating dye such as EvaGreen. During the last decade intercalating dyes have been successfully applied in enzymatic techniques on nucleic acid detection [2–5]. In this paper we chose EvaGreen dye because of the demonstrated contrast between the bright signal it gives off when bound to double-stranded DNA and the quenched fluorescence signal with single-stranded samples [17].

**Fig 1:**
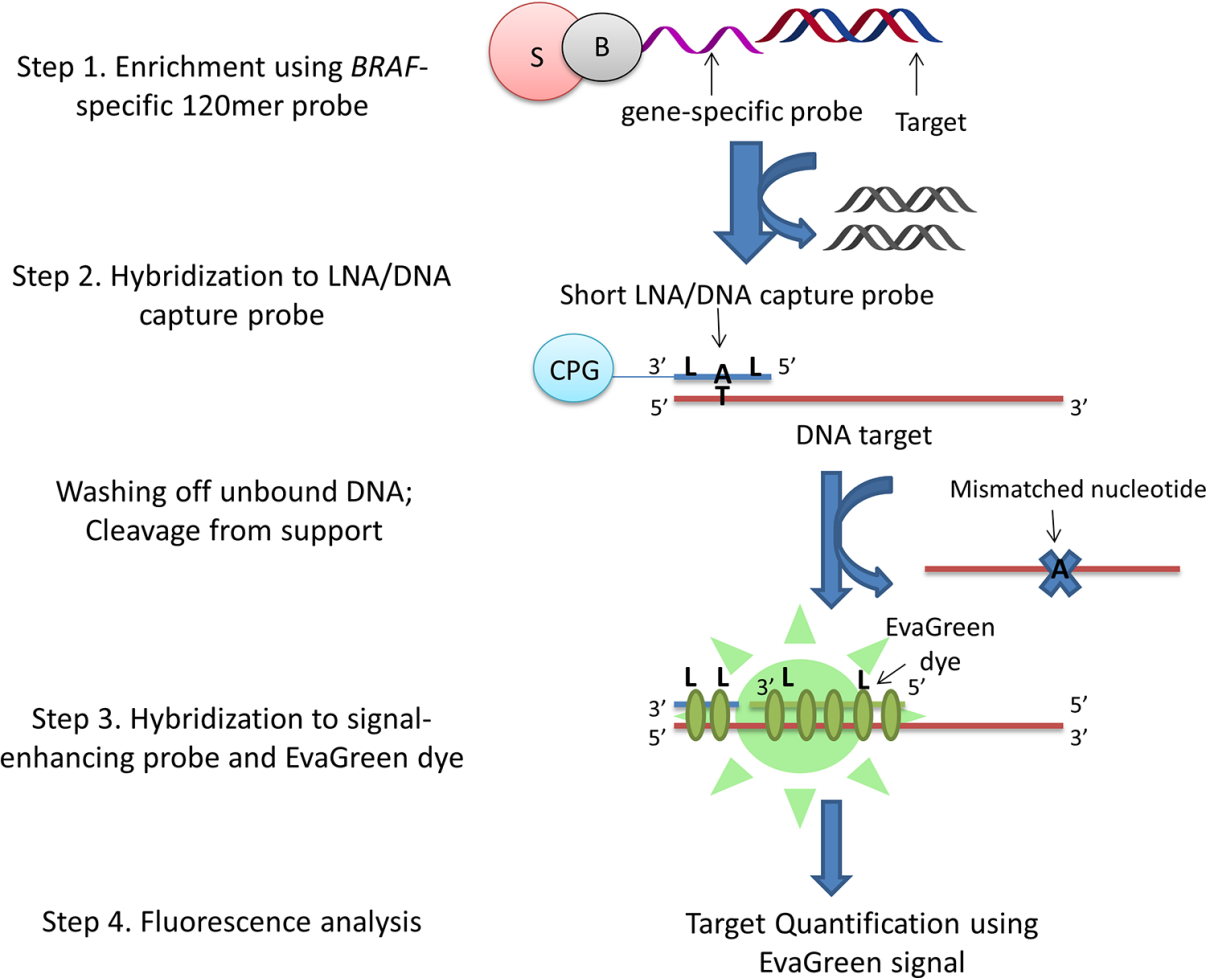
Detection of cancer DNA using novel enzyme-free assay. The main steps of the LNA/DNA assay: 1) binding to gene-specific capture probe, 2) washing and cleavage from support, 3) adding signal-enhancing LNA/DNA probe and intercalating dye, 4) fluorescence detection. B = biotin, S = streptavidin, CPG = controlled pore glass, L = LNA.

Herein, we describe a new assay for rapid enzyme-free detection of *BRAF* V600E mutation (T→A at gene position 1799) in human DNA (Fig 1). We initially enrich the target DNA using a gene specific 120mer enrichment probe, followed by fluorescent detection with EvaGreen dye and a second mutation specific, shorter LNA/DNA capture probe. By using serial dilutions of mutant/wild-type cell line DNA mixtures, we demonstrate that this novel assay is highly potent for the fluorescent sensing of a clinically relevant SNP at low concentrations and high sample complexity. Furthermore we prove that by applying fluorescence microscopy we can specifically detect low femtomolar and attomolar concentrations of DNA containing the target mutation.

## Materials and Methods

### General

Reagents obtained from commercial suppliers were used as directed. LNA Phosphoramidite reagents and EvaGreen dye (20X in water) were obtained from Exiqon and Biotium, respectively. Unmodified and biotinylated DNA strands were purchased from IDT and used after HPLC purification.

Details on oligonucleotide synthesis, characterization and thermal denaturation studies are given in S1 Appendix.

### Genomic DNA

Genomic DNA from cell lines LS411N and HT29 were obtained directly from supplier (ATCC, catalogue nr. CRL-2159 and HTB-38, respectively), and extracted using the Qiagen DNeasy tissue culture extraction system per the manufacturer’s guidelines (Qiagen). HMC-1 (human male control) was obtained from Promega [18]. The product was digested for 12 h at 37°C using EcoRI (New England Biolabs), and precipitated from ethanol. Length of fragments was measured using Agilent BioAnalyzer DNA 7500 kit giving a value of approx. 7000 bp.

#### Pre-enrichment of *BRAF* gene fragment

Pre-enrichment of genomic DNA was carried out using xGen Lockdown kit (IDT), 120mer *BRAF*-specific probe labeled with biotin (IDT), and streptavidin-coated magnetic beads (Dynabeads-M270, LifeTechnologies). **The 120mer probe** was designed to overlap the *BRAF* V600E region (position of V600E mutation is underlined):

5’-ACAACTGTTCAAACTGATGGGACCCACTCCATCGAGATTTCACTGTAGCTAGACCAA

AATCACCTATTTTACTGTGAGGTCTTCATGAAGAAATATATCTGAGGTGTAGTAAGTAAAGG-(biotin-C6)-3’

Pre-digested genomic DNA and biotinylated 120mer probe were incubated for 5 h at 60°C followed by attachment to magnetic beads, multiple washing steps and detachment by heat as suggested by the supplier (IDT; heating to 92°C for 10 min). As a result, single stranded DNA was obtained.

#### Solid-support hybridization assay

The DNA concentration was calculated using the molecular weight and Life Technologies DNA copy number calculator available on their website. Thus, 50 fM of ss DNA fragments (length 5000 nt) corresponded to 3x10^6^ molecules per 100 μL, or 1x10^6^ molecules of the same DNA fragment in the 100 μL of the sample corresponded to the concentration 16.6 fM. Solid support containing corresponding capture probe and target DNA (1 eq.) were placed into 1.5 mL Eppendorf tube containing 100 μL of 1X PBS buffer. The resulting mixture was heated for 10 min at 85°C and subsequently cooled to room temperature over 20 min. The support was centrifuged at 11.000 rpm for 10 min, and after removing the supernate it was washed 2 times with 100 μL 1X PBS at 37°C. Afterwards EvaGreen dye (0.06−0.6X) and the corresponding signal-enhancing probe (4 eq.) were hybridized to the support (100 μL 1X PBS, 85°C, 10 min followed by cooling to RT over 20 min). Fluorescence signal was initially analyzed using standard laboratory UV-vis lamp. For analysis in solution and microscopy, the oligonucleotides were cleaved from support using 32% aqueous ammonia and methylamine 1:1 (v/v) for 4 h at RT, evaporated and re-annealed in presence of EvaGreen dye as described above (0.06−0.6X).

### Fluorometry

DNA amounts (concentrations and number of molecules) were determined using standard UV-spectrophotometry and Life Technologies DNA copy number calculator available as described above. For the annealing, probes were heated for 10 min at 85°C and subsequently cooled to room temperature over 20 min. Fluorometry studies of the resulting samples were performed at 19°C in 1X PBS using PerkinElmer LS 55 luminescence spectrometer equipped with a Peltier Temperature Programmer, excitation at 500 nm and recording emission at 530 nm.

### Fluorescence microscopy

The multiphoton excitation **fluorescence microscopy** measurements were completed on a custom built multiphoton excitation microscope [19]. Briefly, the objective used was a 60X water immersion objective NA 1.29. The laser was a Ti:Sa laser (HPeMaiTai DeepSee, Spectra Physics, Mountain View, CA) and the excitation wavelength used was 840 nm. The fluorescence signals were collected using bandpass filters Bandpass 525/35 nm (AHF Analysentechnik AG, Germany). The detectors were Hamamatsu H7422P-40 PMTs.

## Results and Discussion

Initially, we designed capture probes of different length (9, 11 and 18mers) containing three LNAs in central positions within the sequences (Table 1). According to our design, one of the LNA units was complimentary to the potential *BRAF* V600E mutation (T→A) in the target [16]. We investigated the uniqueness of each capture probe **CP1**-**CP3** for hybridization to the target region as compared to the entire 3 billion bp human genome using in house software available at Stanford University (S1 Table) [20]. Thus, the 9mer and 11mer probes had multiple binding sites in the genome, allowing zero, one or more mismatches, whereas the 18mer probes (**CP3**) were completely specific to the target. As a negative control, we used an equivalent 9mer DNA sequence applied in our previous assays that was not complimentary to the *BRAF* target (capture probe **CP4**) [14].

**Table 1:** Sequences and thermal denaturation temperatures of LNA/DNA capture probes prepared in this study.^a^

Capture probe #	Sequence, 5′→3′	*T*_m_, °C—T1 (Wt)	*T*_m_, °C–T2 (Mut)
**CP1w**	TT+T C+A+C TGT	34.5	22.0
**CP2w**	GAT T+TC +A+CT GT	43.0	33.5
**CP3w**	GAG ATT +TC+A +CTG TAG CTA	56.5	46.0
**CP1m**	TT+T C+T+C TGT	35.0	23.0
**CP2m**	GAT T+TC+T +CT GT	44.0	34.5
**CP3m**	GAG ATT +TC+T +C TG TTA	55.0	46.5
**CP4**	GTG ATA TGC	-	-

^a^ Thermal denaturation temperatures *T*_m_ (°C)/change in *T*_m_ relative to corresponding reference duplex, Δ*T*_m_ (°C). *T*_m_ values measured as the maximum of the first derivatives of the melting curves (A_260_ vs temperature). Reported Tm values were obtained in medium salt buffer and are averages of at least two measurements with resulting *T*_m_ ± 0.5°C. LNA nucleotides are marked with plus before the corresponding letter. Allele-specific nucleotide is underlined. 63Mer *BRAF* target sequences (SNP position is underlined): 5’-CATGAAGACCTCACAGTAAAAATAGGTGATTTTGGTCTAGC TACAGTGAAATCTCGATGGAGT-3’ (**T1**); 5’-CATGAAGACCTCACAGTAAAAATAGGTGATT TTGGTCTAGCTACAGAGAAATCTCGATGGAGT-3’ (**T2**).

Next, **CP1**−**CP4** were prepared using automated solid-phase oligonucleotide synthesis on controlled pore glass (CPG, Table 1). **CP1**−**CP3** were synthesized as both wild-type (w) and mutant (m) variants to *BRAF* 1790–1800 region (i.e. containing nucleotides A and T in the position opposite to the base 1799, respectively). Capture probes were either detached from CPG or kept on solid support after the synthesis (Supporting information). This allowed us to perform the analysis both in solution and on solid support as described below.

We analysed the binding affinities of **CP1**−**CP3** to fully complementary and mismatched 63 nt BRAF fragments in solution (*T*_m_ values, Table 1). As expected, internally positioned LNAs increased the melting temperatures of all the probes by 3.0–4.5°C per one LNA incorporation. Simultaneously target specificity was increased, because the Tm decreased by 10.0–11.0°C in the presence of a mismatch. No duplexes were formed at temperatures above 22°C for the 9mer capture probes **CP1w**,**m**. This suggests that **CP1w**,**m** probes have the highest potential to discriminate a mismatched vs. fully-matched targets. However this might be accompanied by a lack of specificity within the human genome as suggested by our probe uniqueness analysis (Supporting information). In turn, the 18mer capture probes showed high *T*_m_ values for both fully-matched and mismatched duplexes and the 11mer **CP2w** and **CP2m** had somewhat intermediate values (33.5°C–34.5°C in the presence of a mismatch). Finally, negative control **CP4** showed no binding to the target sequences.

**Fig 2:**
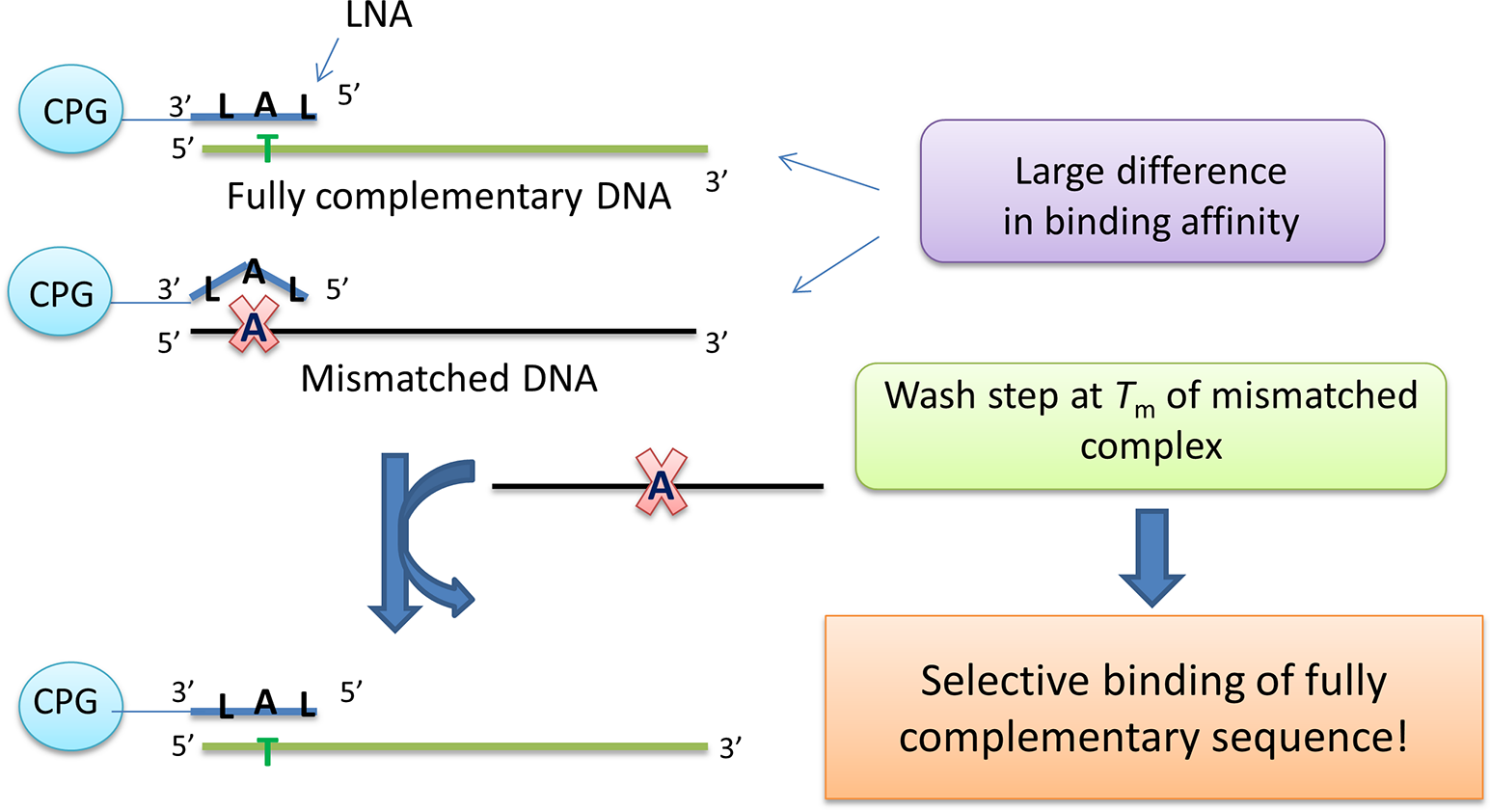
Main principle of DNA detection by short LNA/DNA capture probes on solid support. Target binding specificity results from the difference in melting temperature (*T*_m_) between fully-matched and mismatched capture probe:target complexes. CPG = controlled pore glass.

**Fig 3:**
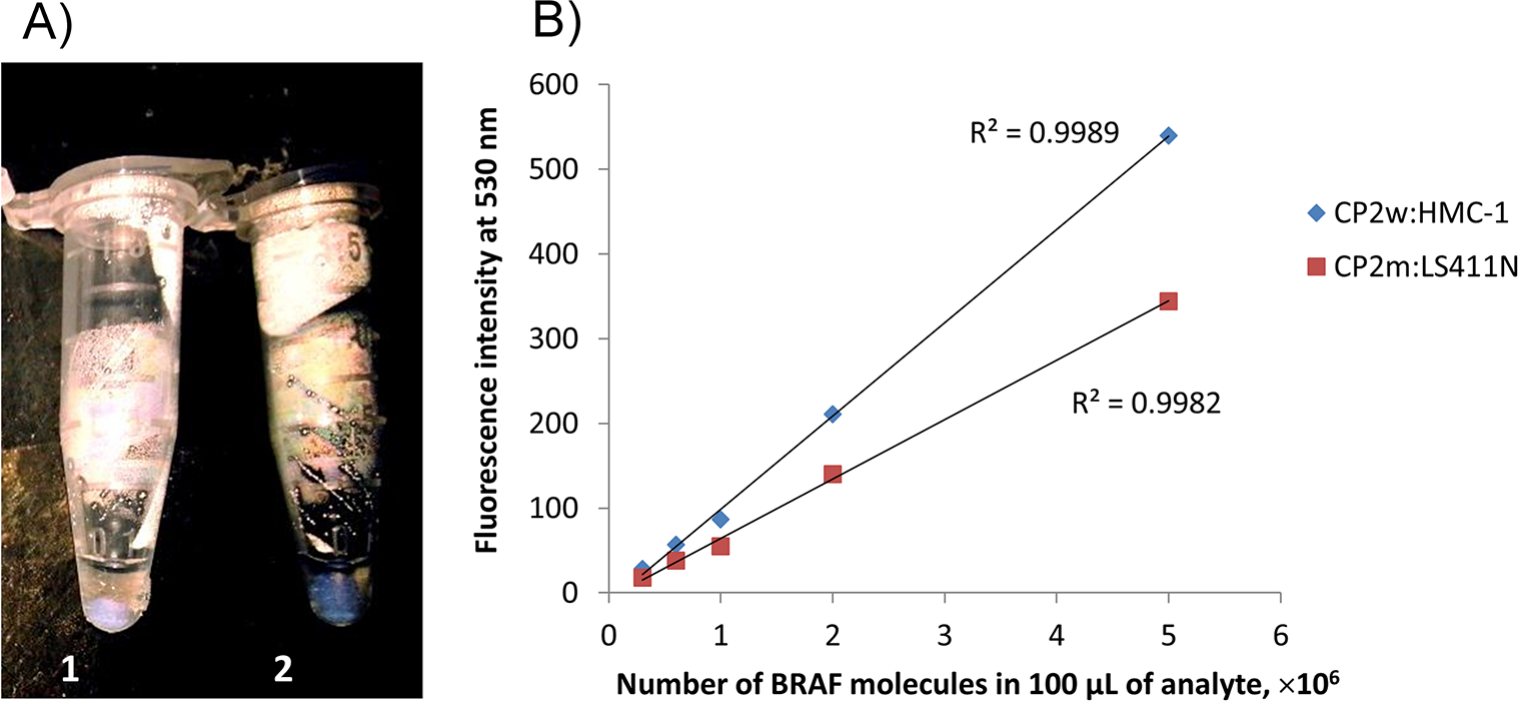
Detection of cancer DNA by fluorescence. (A) Visualization of *BRAF* V600E mutation on solid-support containing capture probe **CP2m**: **CP2m**:HT29 (2.5 pM, tube 1), **CP2m**:LS411N (2.5 pM, tube 6). Signal is obtained under laboratory UV-vis lamp (excitation at 365 nm) at 19°C using 10 pM signal-enhancing probe and 0.6X EvaGreen dye. (B) Quantification of genomic *BRAF* targets by fluorometry in solution. Target titration curves were obtained for fully complementary and mismatched complexes (blue and red lines, respectively) of LNA/DNA capture probes **CP2w** and **CP2m** with corresponding targets and signal-enhancing probe **P1**: 5’-GCT A+GA CCA +AAA TCA CCT A+TT TTT ACT GTG AG+G TCT TCA TGA AGA +AAT AT-3’. LNA nucleotides are marked with plus before the corresponding letter.

Having studied hybridization properties of LNA/DNA capture probes, we applied them to the detection of the *BRAF* V600E mutation in human genomic DNA (Figs 2 and 3, Table 2). We determined, using digital PCR (data not shown), that the human cell lines HT29 and LS411N had a 25.0% and 66.7% abundance of the target mutation, respectively [18]. HMC-1 DNA was used as a 100% wild-type control as it possessed no mutation. First, we pre-enriched the DNA using a 120mer *BRAF* specific probe labeled with streptavidin that did not overlap the region of the capture probe and thus was universal for both the wt and mut targets (Fig 1 and Materials and Methods;). This step increased concentration of the target genome fragment and simultaneously improved specificity of our assay in the same way as when being applied prior to next-generation sequencing [7–8]. After multiple washing steps and detachment from the biotinylated magnetic beads, single-stranded *BRAF* fragments (7000 nt) were recovered into the solution.

**Table 2:** Quantification of *BRAF* V600E mutation in cancer cell line DNA using fluorometry.^a^

Cell line	DNA concentration (wt+mut), fM	I (500/530)–background*		Estimated number of *BRAF* molecules (copy number) per 100 μL, ×10^7^		Abundance of mutant
		CP2w	CP2m	Wt	Mut	sequence, %*
HT29	250	110	23	1.2	0.3	17.3
	50	24	5	0.2	<0.1	17.2
LS411N	250	49	101	0.5	1.0	67.2
	50	10	21	<0.1	0.1	67.7
HMC-1	250	145	-	1.5	0.0	0.0
	50	28	-	0.3	0.0	0.0

^a^ For details of the *BRAF* copy number calculation, see Materials and Methods. Thus, 50 fM ss DNA corresponds to 3x10^6^ molecules per 100 μL, or 1x10^6^ molecules of genomic DNA in 100 μL of the sample corresponds to the concentration 16.6 fM. Abundance value is determined using ratio of EvaGreen fluorescence obtained for individual analysis on wild-type and mutant specific capture probes as follows: I(**CP2m**)/(I(**CP2m**)+I(**CP2w**)).

* I (excitation/emission wavelength, nm); background signal is determined by fluorescence measurement of free single-stranded genomic DNA at the same concentration.

Afterwards, pre-enriched *BRAF* targets were annealed to the capture probes **CP1**–**CP4** directly on solid support [11]. We then used EvaGreen to detect and quantify the double-stranded DNA. EvaGreen signal increases proportionally to the number of base pairs in the formed duplex [17]. That being said, the capture probe:target complex was rather short (9–18 base pairs). In order to avoid a weak signal we applied an additional LNA/DNA signal-enhancing probe complementary to the sequence just adjacent to the capture probe (**P1**; 50 nt). This probe showed no self-folding properties and bound both wild-type and mutant targets universally (Fig 2 and S1 Appendix). Longer signal-enhancing probe would result in even higher signal increase upon target binding. However, LNA/DNA probes of length > 50 nt are challenging to synthesize and purify. Moreover, their target binding properties have to be additionally evaluated to avoid false positive signal due to e.g. self-folding (paper in preparation).

Furthermore, with the solid-support system we were able to take advantage of the *T*_m_ difference between fully matched and mismatched target:probe pairs (Table 1). A simple washing step at the *T*_m_ of the mismatched complex allowed us to eliminate binding of the mismatched sequence. Finally, the sample was re-annealed in the presence of signal-enhancing probe **P1** and EvaGreen dye (Figs 1 and 2).

The solid support containing cancer DNA:probe complex was visualized using a standard laboratory UV lamp (Fig 3 and S2 Table). When the mutant-specific capture probe was applied, the presence of the mutant target could be clearly detected with the naked eye. For further quantification, the sample was detached from CPG, re-annealed and subjected to analysis by fluorometry (Fig 3). We estimated the concentrations of the wild-type and mutant targets in the cancer DNA by using a titration curve. This was generated with a fluorometry assay in solution using a known initial amount of genomic DNA in analyte (in mol and number of molecules), and the same concentrations of reagents as in the experimental assay (see Materials and Methods). Limit of detection (LOD) has been calculated as the lowest target concentration which could be detected with a signal to noise ratio above 3 [14]. Thus, we established LOD value of 50 fM genomic DNA using conventional fluorometry (Table 2; see Materials and Methods for details on the calculation).

When comparing results between capture probes **CP1**−**CP3**, the 11mer **CP2** showed the highest discrimination of mismatched targets while maintaining good binding specificity (Fig 3). In contrast, the longer **CP3** probes showed similar signal for fully matched and mismatched complexes, since the duplex was still formed at the temperature of the assay (Table 1 and S1 Fig). Using **CP2m**, we also achieved high accuracy for the abundance quantification of the cell line LS411N (67.2%–67.7% determined by our assay vs. 66.7% determined by digital PCR). However, the lower mutant abundance in the HT29 DNA was detected with our assay at a higher error level compared to LS411N (deviation of ∼ 8% vs. ≤ 1%, respectively). Most likely, this was caused by the emission signal of the mutant target in HT29 being below our reliable LOD.

The specificity of detection was confirmed by the absence of fluorescence signal when using **CP2m** and wild-type control DNA, HMC-1 (Table 2). Notably, the pre-enrichment step dramatically increased the concentration of *BRAF* fragment in the sample. This was confirmed by up to 10-times elevated non-specific signal upon detection of non-enriched HMC-1 DNA with the **CP2m** probe (data not shown). Therefore, we concluded that both the pre-enrichment step and the design of the mutation probe were essential for the assay. We estimated the accuracy of targeting a specific *BRAF* region to be within ±1%. Taking into account the large sequence complexity of human genomic DNA, we concluded that the accuracy of our *BRAF* gene targeting using LNA/DNA probes was very good [10,21].

**Fig 4:**
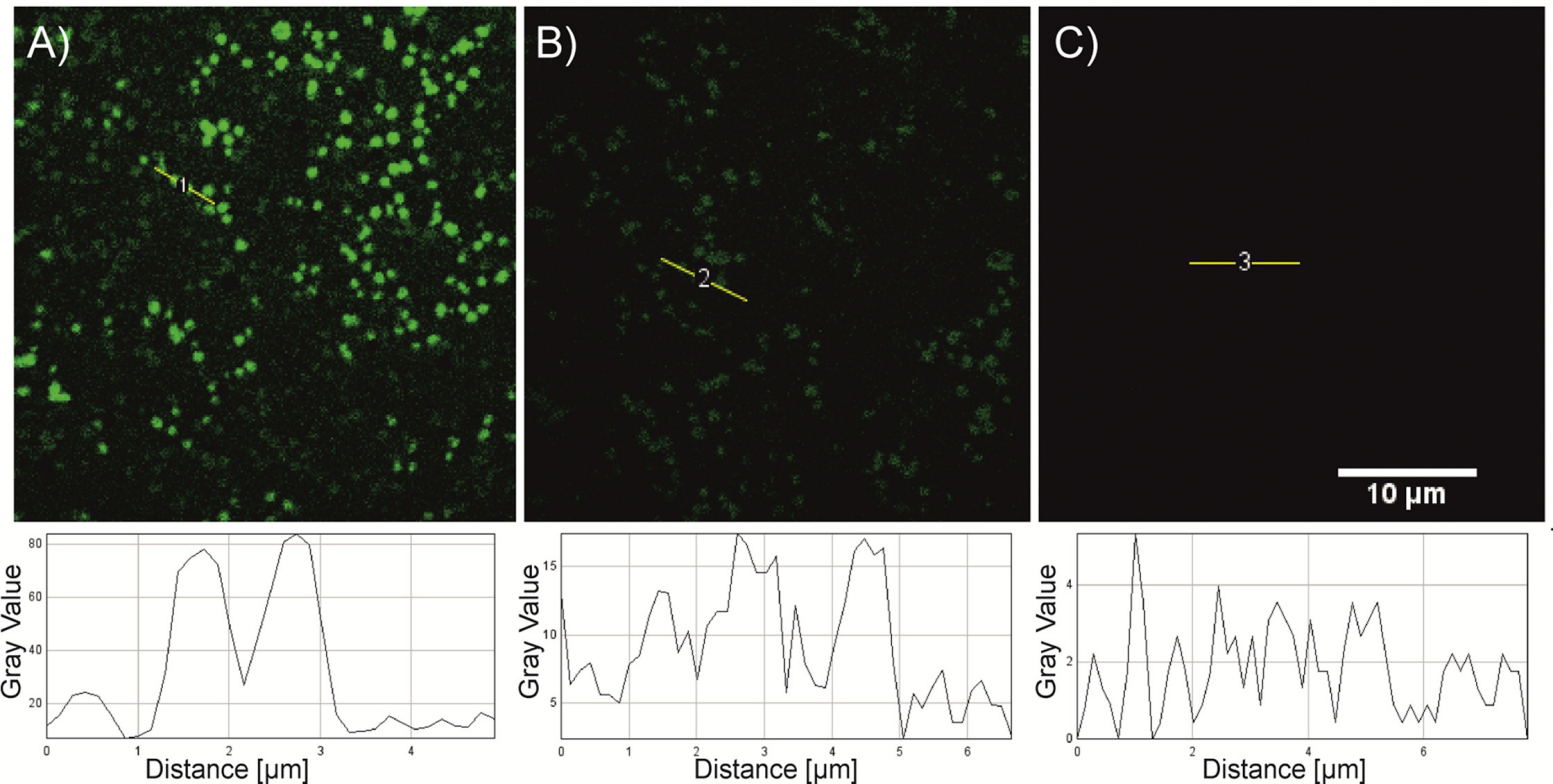
Fluorescence images of *BRAF* DNA fragments from cell line HT29. (A) Complex of DNA with **CP2m**, signal-enhancing probe **P1** and EvaGreen dye, bright dots with intensities over 80 are seen. (B) Target DNA re-annealed with **CP2m** and EvaGreen dye in the absence of **P1**, darker dots are seen with counts up to about 20. (C) EvaGreen dye in 1xX PBS (0.06X solution), no signal is seen. Images were obtained using two photon laser scanning microscope (ex 535+35nm; laser@ 840); at 19°C using 1.5 fM cancer DNA and re-annealing with 10 pM signal-enhancing probe and 0.06X EvaGreen dye. The images were taken using the same instrument settings and adjusted using the same intensity threshold. The graph below the images shows a line plot of the line in the image.

As a final aspect, we subjected a series of highly diluted HT29 mutant DNA molecules to detection by fluorescence microscopy (Fig 4). Prior to analysis, we pre-enriched the target DNA, annealed it to **CP2m** on a solid support (CPG) and afterwards cleaved it from the CPG as described above. With this method the complex of *BRAF* target, **CP2m**, **P1** and EvaGreen dye could be easily detected at ∼ 1.5 fM concentration of mutant DNA corresponding to ∼ 1×10^5^ molecules per 100 μL analyte (for details on calculation, see Materials and Methods). The DNA:EvaGreen complex was seen to form small aggregates on the cover glass surface of approx. 1μm in diameter. In the absence of **P1**, the fluorescence signal was four times dimmer and no fluorescence was observed for the EvaGreen dye control (Fig 4 and S2 Fig). Moreover, no signal was observed using the 100% wild-type control DNA HMC-1 in the same setting. To the best of our knowledge, these LOD values have only been previously reported for PCR-based assays but not any amplification-free methods. Moreover, the observed signal could be detected at 1000-times lower concentrations of DNA than even 1.5 fM corresponding to only 100 molecules per 100 μL analyte. We speculate that by applying this technique and increasing the length of the signal-enhancing probe as mentioned above, the LOD value for cancer DNA could be far below 1 aM.

Comparing our method to previously reported genotyping methods for *BRAF* mutation and other sequence variations, the developed assay is beneficially low in cost, time (12 hours vs. up to 1 week) and robustness [9,14]. Importantly, no enzymes, besides restriction enzymes for the fragmentation genomic DNA, are needed for the detection of the target mutation [22–24]. This prevents errors which often occur with PCR or alignment of sequencing data [3,11]. Stoichiometry of wild type and mutant targets is also conserved in our assay, which allows for an accurate estimation of mutant abundance, given that the limit of target detection requirement is met. In previously reported assays this could be achieved using gold nanoparticles, DNAzymes or electrical detection [25–27]. In this work we improve upon the sensitivity of target detection by applying signal-enhancing probes and laser microscopy, which presents the opportunity to detect sequence variations in target DNA at low concentrations directly in human serum [28].

In summary we describe a novel assay which allows for the rapid analysis of cancer mutations. To achieve necessary specificity and sensitivity of target detection, the assay combines several principles: 1) target pre-enrichment using a long, gene-specific probe (120mer); 2) high binding affinity and specificity of short LNA probes, and 3) DNA detection by high resolution fluorescence microscopy. As we prove in this work, the combination of these techniques enables rapid analysis of mutations in cancer DNA without the need for amplification or other enzymatic reactions. Successfully demonstrated as a proof-of-principle on the V600E mutation in the *BRAF* oncogene, this method can be applied to detect other mutations of clinical significance (e.g. codons 12 and 13 of *KRAS* gene [29]) and serve as a simple, reliable method for research, prognostic or therapy monitoring of clinical samples.

## Supporting Information

S1 Appendix Oligonucleotide synthesis, characterization and thermal denaturation studies. (DOCX)

S1 Fig Visualization of cancer DNA on solid-support containing capture probe CP3m. (Left to right) tubes 1–3: **CP3m**:HT29 (10.0, 5.0 and 2.5 pM), tubes 3–6: **CP3m**:LS411N (10.0, 5.0 and 2.5 pM). Signal is obtained under laboratory UV-vis lamp (excitation at 365 nm), at 19°C using 10 pM signal-enhancing probe and 0.6X EvaGreen dye.
(TIF)

S2 Fig Fluorescence images of *BRAF* DNA fragments from cell line HT29. (A) Complex of DNA with **CP2m**, signal-enhancing probe **P1** and EvaGreen dye, bright dots with intensities over 80 are seen. (B) Target DNA re-annealed with **CP2m** and EvaGreen dye in the absence of **P1**, darker dots are seen with counts up to about 20. (C) EvaGreen dye in 1X PBS (0.06X solution), no signal is seen. (D) Wild-type control DNA HMC-1, no signal is seen.
(TIF)

S1 Table Uniqueness analysis of capture and signal-enhancing probes. (PDF)

S2 Table Colorimetric analysis of model targets and genomic DNA on solid support. (PDF)
