# Supplementary material for: Enzyme-Free Detection of Mutations in Cancer DNA Using Synthetic Oligonucleotide Probes and Fluorescence Microscopy
Source: PLoS One. 2015 Aug 27;10(8):e0136720. doi: 10.1371/journal.pone.0136720 (PMC4552304; doi:10.1371/journal.pone.0136720)
Supplement: S1 Appendix — (DOCX) [file pone.0136720.s001.docx]

**S2 Appendix. Oligonucleotide synthesis, characterization and thermal denaturation studies.**

**Oligonucleotide synthesis** was carried out on Ӓkta Oligopilot DNA synthesizer ([GE Healthcare Life Science](http://www.gelifesciences.com/webapp/wcs/stores/servlet/catalog/en/GELifeSciences-us/products/AlternativeProductStructure_17110/18113679)s) in 200 nmol scale using manufacturer’s standard protocols (DMT-off seting), and 500 Å controlled pore glass (CPG) support (ATDBio). LNA modifications were incorporated automatically (1H-tetrazole activator; 10 min coupling time). The coupling efficiencies of standard DNA phosphoramidites and LNA based on the absorbance of the dimethoxytrityl cation released after each coupling varied between 99% and 100%. CPG was dried in vacuo for 5 min, taken out of column and used directly in solid-phase assays. A portion of CPG support was taken out from the synthetic column, cleaved, deprotected and analysed by ion-exchange (IE) HPLC and MALDI-MS. This allowed us to confirm identity and purity of each capture probe prior to the mutation assay, and have capture probes available for fluorometry assay in solution.

**Cleavage from solid support and removal of nucleobase protecting groups** was performed using 32% aqueous ammonia and methylamine 1:1, v/v, for 4 h at RT. IE HPLC was performed using the Merck Hitachi LaChrom instrument equipped with Dionex DNAPac Pa-100 column (250 mm × 4 mm). Elution was performed starting with an isocratic hold of A- and C-buffers for 2 min followed by a linear gradient to 60% B-buffer over 28 min at a flow rate of 1.0 mL/min (A-buffer: MQ water; B-buffer: 1M NaClO4, C-buffer: 25mM Tris-Cl, pH 8.0). MALDI-TOF mass-spectrometry analysis was performed using a MALDI-LIFT system on the Ultraflex II TOF/TOF instrument from Bruker and using HPA-matrix (10 mg 3-hydroxypicolinic acid, 50 mM ammonium citrate in 70% aqueous acetonitrile).

Purity (%) (MADI MS [M-H]^+^ found/calc.) of capture probes: 92 (2779/2777), **CP1w**; 90 (3419/3419), **CP2w**; 88 (5611/5611), **CP3w**; 94 (2768/2768), **CP1m**; 93 (3409/3410), **CP2m**; 91 (5601/5602), **CP3m**; 87 (2753/2753), **CP4**.

**Thermal denaturation studies** were performed on a Beckman Coulter DU800 UV/VIS spectrophotometer equipped with Beckman Coulter High Performance Temperature Controller in a medium salt phosphate buffer (100 mM NaCl, 10 mM Na-phosphate, 0.1 mM EDTA, pH 7.0). Concentrations of oligonucleotides were calculated using the following extinction coefficients (OD_260_/μmol): G, 10.5; A, 13.9; T, 7.9; C, 6.6. Annealing steps were performed on Eppendorf Thermomixer Shaker equipped with a 0.5 mL block. Oligonucleotides (1.0 μM each strand) were thoroughly mixed, denaturated by heating for 10 min at 85 °C and subsequently cooled to the starting temperature of experiment. Thermal denaturation temperatures (*T*_m_ values, ºC) were determined as maximum of the first derivative of the corresponding thermal denaturation curve (*A* vs. temperature). Reported *T*_m_ values are an average of at least two measurements within ± 0.5 ºC.
