# Supplementary figures and images for: Enzyme-Free Detection of Mutations in Cancer DNA Using Synthetic Oligonucleotide Probes and Fluorescence Microscopy
Source: PLoS One. 2015 Aug 27;10(8):e0136720. doi: 10.1371/journal.pone.0136720 (PMC4552304; doi:10.1371/journal.pone.0136720)

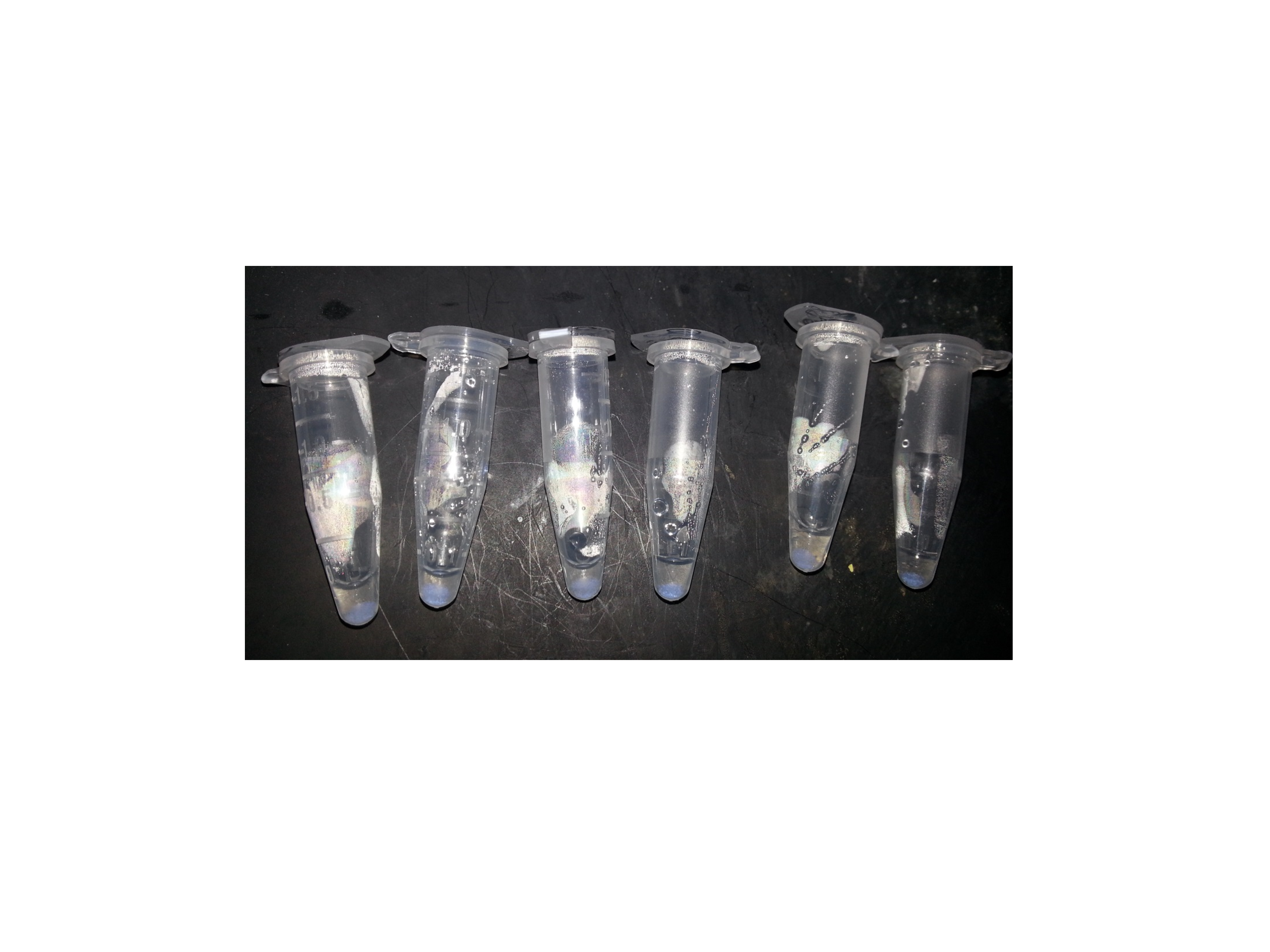

Supplement: S1 Fig — (Left to right) tubes 1–3: CP3m:HT29 (10.0, 5.0 and 2.5 pM), tubes 3–6: CP3m:LS411N (10.0, 5.0 and 2.5 pM). Signal is obtained under laboratory UV-vis lamp (excitation at 365 nm), at 19°C using 10 pM signal-enhancing probe and 0.6X EvaGreen dye. (TIF) [file pone.0136720.s002.tif]

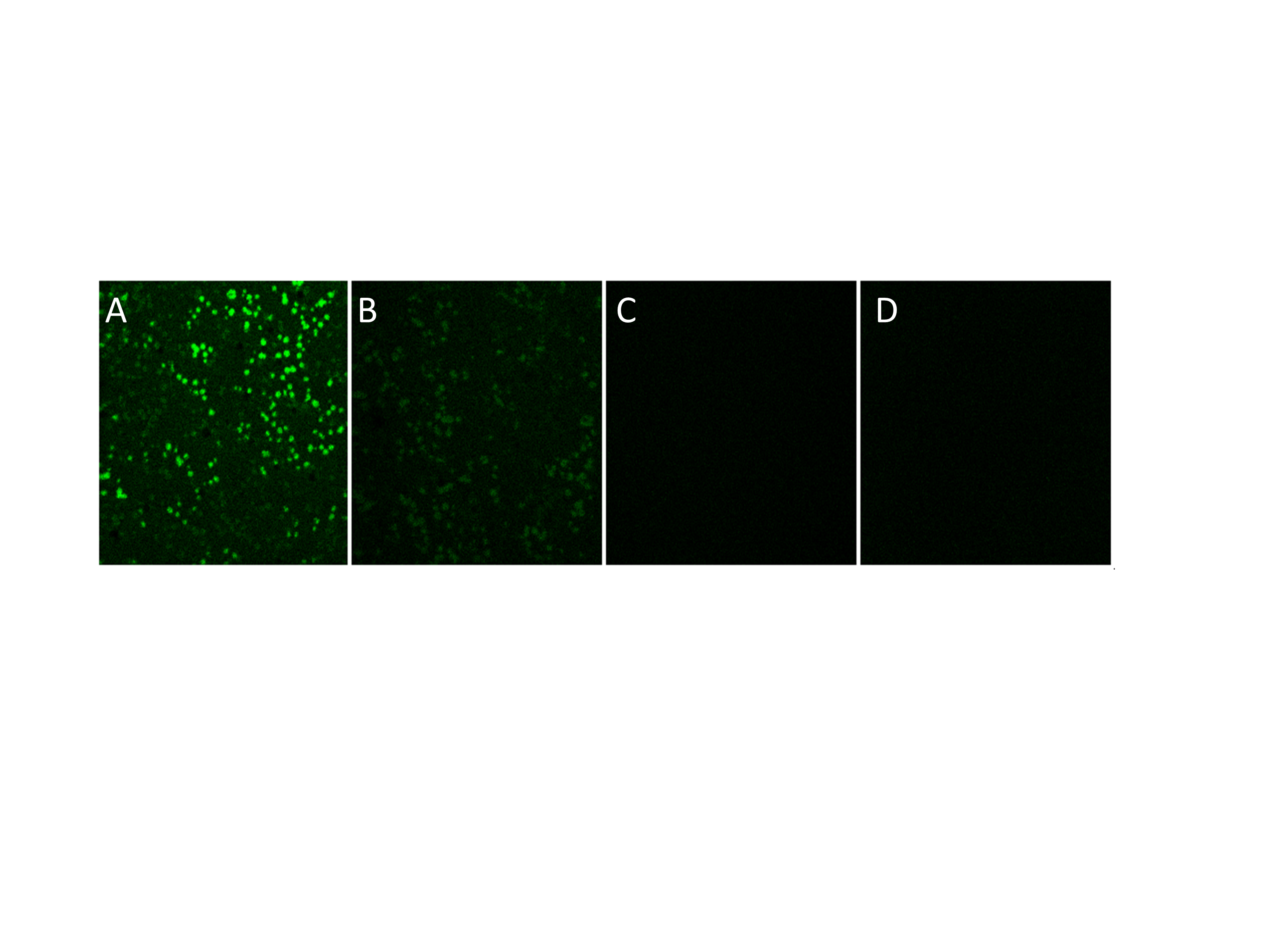

Supplement: S2 Fig — (A) Complex of DNA with CP2m, signal-enhancing probe P1 and EvaGreen dye, bright dots with intensities over 80 are seen. (B) Target DNA re-annealed with CP2m and EvaGreen dye in the absence of P1, darker dots are seen with counts up to about 20. (C) EvaGreen dye in 1X PBS (0.06X solution), no signal is seen. (D) Wild-type control DNA HMC-1, no signal is seen. (TIF) [file pone.0136720.s003.tif]
