# Supplementary material for: Enzyme-Free Detection of Mutations in Cancer DNA Using Synthetic Oligonucleotide Probes and Fluorescence Microscopy
Source: PLoS One. 2015 Aug 27;10(8):e0136720. doi: 10.1371/journal.pone.0136720 (PMC4552304; doi:10.1371/journal.pone.0136720)
Supplement: S1 Table — (PDF) [file pone.0136720.s004.pdf]

S1 Table. Uniqueness analysis of capture and signal-enhancing probes.[a]

Probe #	Sequence,* 5'→3'	Number of potential target sequences in human genome:		
		perfect match	1 mismatch**	2 mismatches
**CP1w**	TTTCACTGT	95.014	N/A***	N/A
**CP2w**	GATTTCACTGT	3.999	N/A	N/A
**CP3w**	GAGATTTCACTGTAGCTA	1	2 (13,18)	3(1,18|17-18|17-18)
**CP1m**	TTTCTCTGT	114.261	N/A	N/A
**CP2m**	GATTTCTCTGT	4.686	N/A	N/A
**CP3m**	GAGATTTCTCTGTAGCTA	0	4 (1;18;1;1,18)	>7
**P1**	GCT AGA CCA AAA TCA CCT ATT TTT ACT GTG AGG TCT TCA TGA AGA AAT AT	1	0	0

[a] Numbers in parentheses indicate the position of mismatches in the sequences. The analysis of short capture sequences (9 nt and 11 nt) was performed determining the presence of this sequence from the human genome reference (hg19). For longer sequences (18 nt, 50 nt and 53 nt), we applied BLASTN via NCBI website. * The sequences are analyzed in absence of LNA modifications.

**"1 mismatch" indicates the number of compliments in the human genome if one base of the sequence is subsituted.

*** In the case of sequences with a high number of perfect matches, we did not check the amount of matches if 1 or more bases of the sequence were substituted.
