# Supplementary material for: Enzyme-Free Detection of Mutations in Cancer DNA Using Synthetic Oligonucleotide Probes and Fluorescence Microscopy
Source: PLoS One. 2015 Aug 27;10(8):e0136720. doi: 10.1371/journal.pone.0136720 (PMC4552304; doi:10.1371/journal.pone.0136720)
Supplement: S2 Table — (PDF) [file pone.0136720.s005.pdf]

S2 Table. Colorimetric analysis of model targets and genomic DNA on solid support.[a]

Capture Probe #	Color Reference target:			Color Cell line DNA:	
	Control (Water)	**T1** (Wt 63 nt)	**T2** (Mut 63 nt)	*LS411N*	*HT29*
**CP1w**	-	++	+	++	+++
**CP2w**	-	+++	-	++	++++
**CP3w**	-	+++	+	++	++++
**CP1m**	-	+	+	++	+
**CP2m**	-	-	++	++	+
**CP3m**	-	++	++++	++	+
**CP4**	-	-	-	-	-

[a] Conditions of measurements: excitation wavelength = 365 nm; approx. 33 nmol capture on CPG support (one sixth part of the 200 nmol oligonucleotide synthesis load); target and signal-enhancing probe (2.5 pM and 10 pM, respectively); 0.6X EvaGreen dye in 100 μM 1X PBS. For sequences **T1** and **T2**, see Table 1.
